# Mesalazine-Induced Myocarditis in Inflammatory Bowel Disease: A Systematic Review

**DOI:** 10.7759/cureus.78208

**Published:** 2025-01-29

**Authors:** Kiran Jhakri, Moath Al-Shudifat, Bushra Sumra, Cyril Kocherry, Hina Shamim, Lubna Mohammed

**Affiliations:** 1 Internal Medicine, Shahjalal University of Science and Technology, Sylhet, BGD; 2 Internal Medicine, Faculty of Medicine, Cairo University, Cairo, EGY; 3 Clinical Research, Sanmora Bespoke Clinical Research Solutions, East Orange, USA; 4 School of Medicine, Ninewells Hospital, Dundee, GBR; 5 Pediatrics, Baqai Medical University, Karachi, PAK; 6 Internal Medicine, Dr. Vizarath Rasool Khan (VRK) Women's Medical College, Hyderabad, IND

**Keywords:** crohn's disease, inflammatory bowel disease, mesalamine, mesalazine, myocarditis, ulcerative colıtıs

## Abstract

Crohn's disease (CD) and ulcerative colitis (UC) are two forms of inflammatory bowel disease (IBD). This chronic, immune-mediated disorder leads to inflammation in specific gastrointestinal tract regions. Myocarditis is a rare but significant IBD complication that affects roughly 0.3% of cases. Mesalazine-induced myocarditis is a rare side effect of mesalazine therapy, which is considered a standard treatment for IBD. Increased mortality and cardiogenic shock are possible outcomes of this adverse response. The objectives of this study are to characterize the clinical features of mesalazine-induced myocarditis in patients with IBD, to conduct a comprehensive analysis of mesalazine-related myocarditis cases in IBD patients, to review the existing literature, to elucidate the pathophysiological mechanisms of myocarditis in IBD, and to determine whether myocarditis represents an extraintestinal manifestation of IBD or an adverse drug reaction to mesalazine. This systematic review followed the Preferred Reporting Items for Systematic Reviews and Meta-Analyses (PRISMA) 2020 guidelines. Relevant literature was retrieved from Cochrane, ScienceDirect, Google Scholar, PubMed, and PubMed Central (PMC). Only articles published in English or with a full English translation available within the last 10 years (2014-2024) were included. A rigorous quality assessment tool was applied to ensure the quality of evidence-based medicine that will be utilized to construct a conclusion and direct future reviews. Among 43 patients analyzed, 29 (67%) developed myocarditis attributable to mesalazine treatment, while 14 (33%) exhibited myocarditis unrelated to the medication. Our findings indicate that myocarditis in IBD is more likely to be a severe drug reaction than an extraintestinal manifestation of IBD progression. In drug-induced myocarditis cases, mesalazine derivatives, including sulfasalazine, mesalamine, and balsalazide, were most frequently implicated. Potential mechanisms underlying mesalazine-associated myocarditis include IgE-mediated hypersensitivity reactions, direct cardiotoxicity, cell-mediated hypersensitivity, or humoral antibody responses to drug metabolites. When treating myocarditis in IBD, whether due to medication or as an extraintestinal manifestation, discontinuing the offending drug and initiating immunosuppressive therapy appear to be the most effective approach.

## Introduction and background

Mesalazine, sometimes referred to as mesalamine or 5-aminosalicylic acid (5-ASA), and its derivatives are essential components of maintenance therapy for achieving disease remission in inflammatory bowel disease (IBD). 5-ASA compounds are widely used in the treatment of newly diagnosed IBD despite their more limited efficacy in Crohn's disease (CD) than in ulcerative colitis (UC) [1]. Mesalazine's well-established anti-inflammatory qualities make it the recommended treatment for IBD. The active byproducts of sulfasalazine are 5-ASA and sulfapyridine. In contrast to sulfasalazine, mesalazine is made entirely of 5-ASA, lacks the sulfapyridine carrier molecule, and has no sulfur component [2]. 5-ASA is thought to function by blocking the enzymes lipoxygenase and cyclooxygenase, which lowers the synthesis of leukotrienes and pro-inflammatory prostaglandins. However, the precise mechanism is still unknown [3].

Furthermore, 5-ASA has the ability to scavenge free radicals, function as an antioxidant, and perhaps activate the peroxisome proliferator-activated receptor-γ (PPAR-γ) [3]. This activation can reduce signaling through the PPAR-γ pathway, subsequently lowering nuclear factor κB (NF-κB) activity. Mesalazine helps mitigate colonic inflammation by interrupting this inflammatory cascade. When administered orally, mesalazine is primarily absorbed in the stomach, resulting in localized effects [4]. The majority of unpleasant reactions, such as nausea, vomiting, diarrhea, and abdominal discomfort, are therefore restricted to the gastrointestinal tract, and systemic side effects are very uncommon. But it's important to understand that, even if they are uncommon, mesalazine can cause side effects that go beyond the gastrointestinal system. These less frequent adverse effects include blood dyscrasias, pancreatitis, and cardiovascular complications [4].

IBD affects about 1.5 million people at the moment [5]. IBD is characterized by inflammation of various parts of the gastrointestinal system and encompasses a group of chronic, immune-mediated disorders, including CD and UC. Although the pathogenesis of IBD is not yet fully understood, it is believed to result from alterations in the human microbiome, dysregulation of the immune system, and a complex interplay of genetic and environmental factors [6]. In addition to gastrointestinal involvement, IBD is associated with a range of extraintestinal manifestations, frequently affecting the musculoskeletal, dermatological, hepatic, pancreatic, biliary, ophthalmic, renal, and pulmonary systems [5]. Cardiac extraintestinal manifestations, including pericarditis, myocarditis, arrhythmias, and heart failure, have been reported, although they are rare. Among these, myocarditis is the most prevalent cardiovascular complication, accounting for 70% of all IBD-related heart issues [5].

Myocarditis is an inflammatory condition of the heart muscle that can be recognized by immunological, histological, and immunohistochemical criteria [7]. A rare extraintestinal side effect of IBD that affects about 0.3% of patients is myocarditis. Though less common, mesalazine-induced myocarditis is a known side effect that can result in cardiogenic shock and death [8]. With a 0.04% incidence rate, myocarditis is one of the infrequent extraintestinal symptoms of UC that might be challenging for treating physicians to diagnose [9]. Understanding the inflammatory processes and their relationship with 5-ASA therapy is critical to preventing further morbidity and mortality. Early use of sulfasalazine suggested that the sulfapyridine component of the drug was responsible for this drug-induced inflammation. However, subsequent experiences with balsalazide and 5-ASA alone (mesalazine) have shown that 5-ASA itself can trigger inflammation [1]. Although the exact cause of the inflammation remains unclear, one of the four hypothesized mechanisms is thought to be responsible: a direct toxic effect on the myocardium or pericardium, an immunoglobulin E (IgE)-mediated allergic reaction, a cell-mediated hypersensitivity reaction, or a humoral antibody response [1].

In most cases, discontinuing the 5-ASA product is sufficient to alleviate symptoms and confirm a diagnosis of drug-induced myocarditis [10]. Multiple perspectives exist on the etiology of myocarditis in IBD. A better understanding of the pathophysiological mechanisms behind extraintestinal cardiac complications can help prevent these issues and improve treatment outcomes for future patients. Developing a systematic therapeutic approach to treat myocarditis in patients with IBD can improve overall patient outcomes, prevent delays in appropriate treatment, and minimize the severity of myocarditis [5].

The objectives of our study are to describe the clinical features of patients diagnosed with mesalazine-induced myocarditis in IBD, conduct a comprehensive analysis of cases of mesalazine-induced myocarditis in individuals with IBD, evaluate the existing body of data, elucidate the pathophysiology of myocarditis in IBD, and determine whether myocarditis is more an extraintestinal manifestation or an adverse effect of mesalazine.

## Review

Method

To conduct this systematic assessment, we followed the 2020 recommendations of the Preferred Reporting Items for Systematic Reviews and Meta-Analyses (PRISMA).

Search Method and Sources Used

We searched relevant articles on PubMed, PubMed Central (PMC), Google Scholar, ScienceDirect, and Cochrane. Table 1 indicates the search method, database used, and number of results found on each database.

**Table 1:** Articles identified using each database and search strategy used for each database. MeSH: Medical Subject Headings; PMC: PubMed Central

Strategy used	Database	Number of articles identified
myocarditis and mesalazine and inflammatory bowel disease	PubMed	4
Mesalazine OR ( "Mesalamine/adverse effects"[Mesh] OR "Mesalamine/classification"[Mesh] OR "Mesalamine/pharmacokinetics"[Mesh] OR "Mesalamine/toxicity"[Mesh] ) AND Myocarditis OR ( "Myocarditis/complications"[Mesh] OR "Myocarditis/etiology"[Mesh] OR "Myocarditis/physiopathology"[Mesh] )AND Inflammatory Bowel Disease OR ( "Inflammatory Bowel Diseases/classification"[Mesh] OR "Inflammatory Bowel Diseases/complications"[Mesh] OR "Inflammatory Bowel Diseases/diagnosis"[Mesh] OR "Inflammatory Bowel Diseases/etiology"[Mesh] OR "Inflammatory Bowel Diseases/physiopathology"[Mesh] OR "Inflammatory Bowel Diseases/prevention and control"[Mesh] )	PubMed (MeSH)	416
Mesalazine induced Myocarditis in Inflammatory Bowel Disease	PMC	336
Mesalazine induced Myocarditis in Inflammatory Bowel Disease	Google Scholar	210
Mesalazine induced Myocarditis in Inflammatory Bowel Disease	ScienceDirect	52
Mesalazine AND Inflammatory bowel disease AND Myocarditis	Cochrane	26
Total		1044

Inclusion and Exclusion Criteria

We covered articles written in English and published within the last 10 years (2014-2024) or those with an available English translation. The study focuses on male and female patients between the ages of 19 and 44 who were diagnosed with myocarditis. Exclusion criteria include patients younger than 19 or older than 44, articles not published in English, preprints, and animal studies.

Selection Process

The EndNote library (Clarivate, London, United Kingdom) was updated with all relevant articles, and duplicates were removed. We then screened the titles and abstracts to filter eligible papers. The shortlisted papers were evaluated for full-text availability. After selecting pertinent full-text articles, articles that did not fulfill the inclusion and exclusion criteria were eliminated.

Quality Appraisal of the Selected Articles

We evaluated all shortlisted papers using appropriate quality assessment tools tailored to the type of research. The quality assessment of case reports was assessed using the Joanna Briggs Institute (JBI) checklist, and systematic review was evaluated using the Assessment of Multiple Systematic Reviews (AMSTAR 2) tool [11,12]. Only papers that met the quality appraisal criteria were included in this systematic review.

Results

Study Identification and Selection

**Figure 1:**
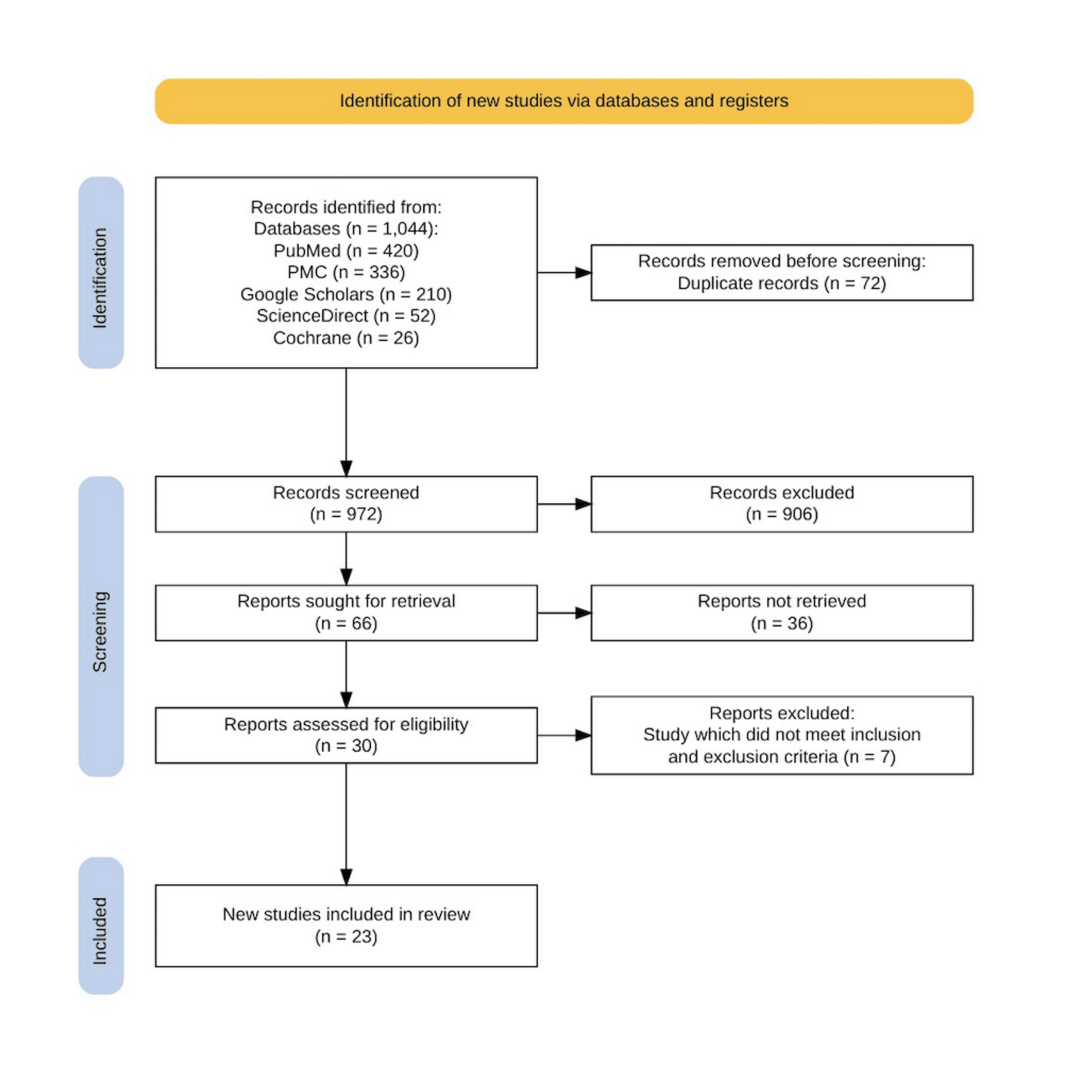
PRISMA flow diagram showing the data selection process. PRISMA: Preferred Reporting Items for Systematic Reviews and Meta-Analyses; PMC: PubMed Central

Using multiple databases, including PubMed, PMC, Google Scholar, ScienceDirect, and Cochrane, we identified 1,044 relevant papers. After importing all selected articles into EndNote, 72 duplicate records were removed. Screening of the remaining paper's titles and abstracts resulted in 66 articles being selected for further consideration. Following a full-text review of these shortlisted articles, 30 papers were evaluated for eligibility and quality using appropriate quality appraisal tools. Ultimately, 23 articles were selected for in-depth review. The PRISMA flowchart, illustrating the selection process, is depicted in Figure 1.

Quality Appraisal

The appropriate tools for quality appraisal were used to evaluate the final papers. Table 2 shows the quality appraisal results using the JBI tool [11]. 

**Table 2:** Quality appraisal results using the JBI tool. +: yes; -: no; ?: unclear; JBI: Joanna Briggs Institute

JBI critical appraisal checklists	Chaudhry et al. [2]	Song and Seo [3]	Kyriakou et al. [4]	Ali et al. [8]	Littlewood et al. [9]	Dias et al. [10]	Asadi et al. [13]	Caio et al. [14]	Lee and Baek [15]	Okoro et al. [16]	Roczek et al. [17]	Baker et al. [18]	Gruenhagen et al. [19]	Ibrahim et al. [20]	McGrath-Cadell et al. [21]	Fleming et al. [22]	Kim et al. [23]	Piazza et al. [24]	Shergill [25]	Cho et al. [26]	Daloya et al. [27]	Bernardo et al. [28]
1. Were patient's demographic characteristics clearly described?	+	+	+	+	+	+	+	+	+	+	+	+	+	+	+	+	+	+	+	+	+	+
2. Were a timeline and a detailed description of the patient's history provided?	+	+	+	+	+	+	+	+	+	+	+	+	+	+	+	+	+	+	+	+	+	+
3. Was the patient's clinical state at the time of presentation adequately described?	+	+	+	+	+	+	+	+	+	+	+	+	+	+	+	+	+	+	+	+	+	+
4. Were the results of any diagnostic tests or evaluation techniques explained in detail?	+	+	+	+	+	+	+	+	+	+	+	+	+	+	+	+	+	+	+	+	+	+
5. Did the therapy technique or interventions provide a clear description?	+	+	+	+	+	+	+	+	+	+	+	+	+	+	+	+	+	+	+	+	+	+
6. Was a detailed description of the clinical state following the intervention?	+	+	+	+	+	+	+	+	+	+	+	+	+	?	+	+	?	+	_	_	?	+
7. Did adverse occurrences (harms) or unexpected events become recognized and documented?	_	?	_	?	+	-	_	_	+	?	+	-	_	+	?	_	_	?	?	?	_	?
8. Are there any lessons to be learned from the case report?	?	+	+	+	+	+	?	+	?	_	_	+	_	_	+	?	+	_	+	_	?	_

Table 3 shows the quality appraisal results using the AMSTAR 2 tool [12]. 

**Table 3:** Quality appraisal using the AMSTAR tool. +: yes; -: no; AMSTAR: Assessment of Multiple Systematic Reviews; RoB: risk of bias; PICO: population, intervention, comparison, and outcome

AMSTAR 2 criteria	Giordani et al. [6]
Components of PICO	+
Review procedures that have been implemented and any significant protocol deviation	+
Rationale for the choice of research designs	+
An explanation of the literature search approach	+
Selection of a duplicate study was made	+
The extraction of duplicate data was carried out	+
An explanation of the excluded studies was given	+
A thorough explanation of the included research	+
Evaluation of individual studies' RoB	+
Reporting on the sources of financing	+
Using the right techniques for the statistical combining of results	+
RoB's effect in individual studies on the meta-analysis's findings	+
RoB was utilized to interpret the findings	+
Reasons for the results' heterogeneity	+
Examining publication bias and how it affects the outcomes	+
Financial and conflicting interests	-

Table 4 provides a summary of the overall study. Diagnosis of the individual cases, intervention applied, duration of onset of symptoms, and conclusion of each case are mentioned briefly.

**Table 4:** Summary table about the various treatment options for myocarditis in IBD and the measures used in each study. 5-ASA: 5-aminosalicylic acid; AZA: azathioprine; ECMO: extracorporeal membrane oxygenation; g: gram; IV: intravenous; IgG: immunoglobulin G; kg: kilogram; mg: milligram; IBD: inflammatory bowel disease; UC: ulcerative colitis; CD: Crohn's disease

Serial no.	Author	Type of study	Age and gender of the patient	Year of publication	Diagnosis	Intervention	Onset of symptoms of myocarditis after mesalazine	Conclusion
1	Chaudhry et al. [2]	Case report	32 years old, male	2023	CD	Infliximab 400 mg (5 mg/kg) and mesalazine 1 g thrice daily	After 1 week	After stopping mesalazine, the patient's symptoms improved
2	Song and Seo [3]	Case report	31 years old, male	2022	UC	5-ASA 2.4 g daily	After 20 days	After 5-ASA was stopped, methylprednisolone 60 mg/day, human IgG 0.5 g/kg, and antibiotics (levofloxacin and metronidazole) were administered
3	Kyriakou et al. [4]	Case report	32 years old, male	2023	UC	Mesalazine 1.5 g twice daily	After 12 days	The patient's symptoms improved after stopping mesalazine
4	Giordani et al. [6]	Systematic review	Median age, 31 years old (both male and female)	2023	UC and CD	5-ASA-derived drugs	Within 30 days after the initiation of drugs	Symptoms improved after mesalamine was stopped
5	Ali et al. [8]	Case report	19 years old, female	2021	UC	Mesalazine 2.4 g plus oral prednisolone (starting at 30 mg once daily) was given	After 4 weeks	Mesalazine was ceased. The symptoms were alleviated by infliximab and analgesics
6	Littlewood et al. [9]	Case report	28 years old, male	2024	UC	Mesalazine	After 3 weeks	The symptoms were improved after mesalazine was stopped and prednisolone was started
7	Dias et al. [10]	Case report	19 years old, male	2018	UC	Mesalazine 1500 mg twice daily	After 2 weeks	Mesalazine was ceased. Vedolizumab was administered to the patient since AZA and infliximab failed to alleviate their symptoms
8	Asadi et al. [13]	Case report	25 years old, male	2018	UC	Mesalazine 2 g twice daily	Symptoms for 2 weeks while on remission therapy with mesalazine	The patient's symptoms improved after stopping mesalazine
9	Caio et al. [14]	Case report	26 years old, male	2021	UC	Initial intervention after the diagnosis of UC: oral mesalazine (4.8 g/day) and beclomethasone (5 mg/day)	In this patient, symptoms of myocarditis were not associated with mesalazine therapy	Even after stopping mesalazine, the patient's myocarditis symptoms returned, indicating that the medication was not the cause. IV vedolizumab 300 mg every 8 weeks, bisoprolol 2.5 mg, and ramipril 5 mg daily helped the patient's symptoms
						1st exacerbation: mesalazine was stopped		
						AZA 150 mg/day associated with methylprednisolone 1 mg/kg/day for 7 days, tapered down until discontinuation		
						2nd exacerbation: AZA was stopped, and vedolizumab was started		
10	Lee and Baek [15]	Case report	29 years old, female	2024	UC	5-ASA 4,800 mg per day	After 21 days	The patient's symptoms improved after 5-ASA treatment was stopped and IV steroids (methylprednisolone) and antibiotics (tazobactam/piperacillin) were administered
11	Okoro et al. [16]	Case report	23 years old, male	2018	UC	At the initial diagnosis of UC: mesalazine 800 mg thrice daily along with 60 mg of prednisone	Symptoms of myocarditis appeared after 6 months of the initial diagnosis of UC	Mesalazine was discontinued, methylprednisolone was administered, and adalimumab was initiated
						2nd regimen: 2.4 mg daily		
						3rd regimen after acute exacerbation: 4.8 mg daily		
12	Roczek et al. [17]	Case report	26 years old, male	2014	UC	Mesalazine 500 mg thrice daily and prednisone 50 mg once daily	After 2 weeks	After stopping mesalazine, the patient's symptoms improved
13	Baker et al. [18]	Case report	38 years old, male	2015	CD	Mesalazine delayed-release capsules (Delzicol) 800 mg three times/day (2.4 g/day) and a mesalamine suppository (Canasa) 1000 mg at bedtime	After 3 weeks	After stopping mesalazine, the patient's symptoms improved. 6-Mercaptopurine and infliximab were introduced to treat CD
14	Gruenhagen et al. [19]	Case report	24 years old, male	2014	UC	Not mentioned	Symptoms occurred as an extraintestinal manifestation of UC after 3 months	Daily oral prednisone and mesalazine therapies improved both cardiac and gastrointestinal symptoms
15	Ibrahim et al. [20]	Case report	21 years old, male	2019	CD	Mesalazine daily	After 4 weeks	After stopping mesalazine, the patient's symptoms subsided
16	McGrath-Cadell et al. [21]	Case report	27 years old, female	2020	CD	Prednisolone was weaned by 5 months post-discharge, and AZA 175 mg and colchicine were continued	Myocarditis relapse occurred after 7 months	The patient was kept under anticoagulant and immunosuppression therapy
17	Fleming et al. [22]	Case report	31 years old, male	2015	UC	High-dose oral mesalazine (2.4 g twice daily)	After 3 days	Mesalazine was stopped. In this patient, symptoms further deteriorate into cardiogenic shock. Ciclosporin was given for UC
18	Kim et al. [23]	Case report	28 years old, female	2016	UC	ECMO for cardiac resting and methylprednisolone 33 mg	Not mentioned	In this patient, myocarditis occurred as an extraintestinal manifestation of UC and was managed by ECMO and infliximab for uncontrolled inflammation by corticosteroid
19	Piazza et al. [24]	Case report	22 years old, male	2022	UC	5-ASA 1600 mg twice daily and systemic steroid (prednisone 40 mg IV once daily)	Acute myocarditis was presented as the first symptom of UC	5-ASA was stopped due to hepatic and pancreatic toxicity, and 75 mg of AZA was started instead
20	Shergill [25]	Case report	22 years old, male	2021	UC	Mesalazine 1.6 g twice daily and prednisolone 40 mg	After 12 days	Mesalazine was stopped and patient symptoms were improved
21	Cho et al. [26]	Case report	32 years old, male	2022	UC	Mesalazine 4.8 g four times daily and prednisone enemas	After 13 days	After stopping mesalazine, the patient's symptoms subsided
22	Daloya et al. [27]	Case report	34 years old, male	2022	CD	Mesalazine intermittently	Not mentioned	Therapy with adalimumab was initiated. It was believed that the autoimmune process, an extraintestinal sign of CD, was the cause
23	Bernardo et al. [28]	Case report	20 years old, female	2016	UC	Mesalazine oral (3 g/day) and topical (3 g/week)	After 2 weeks	The patient's symptoms improved once mesalazine was stopped

Overall Population Analysis

We conducted a descriptive analysis of all the population's findings. Forty-three patients made up the total population (22 from the individual case reports and 21 from the single-center cohort conducted by Giordani et al. in their systematic review of the literature). With a median age at diagnosis of 31 years (IQR 19-44), the majority of patients (77%) were male and Caucasian (53%; just two patients were Asian). Of these, 85% were on medications derived from 5-ASA, 58% had UC, and 30% had CD. These drugs were started less than a month before the development of cardiovascular symptoms in 82% of cases. Heart failure (14%) and arrhythmias (14%) were less common clinical manifestations of myocarditis than infarct-like chest discomfort (67%). Notably, no cardiovascular symptoms were reported by two patients (5%) in this study. Most cases had elevated levels of troponin I and C-reactive protein (CRP). The median left ventricular ejection fraction (LVEF) (IQR 31-66) was 45%. However, at diagnosis, left ventricular (LV) dysfunction (LVEF <50%) was seen in 35% of patients. Thirty-three patients had cardiac magnetic resonance (CMR), and in most cases (77%), the results showed late gadolinium enhancement (LGE) and, in 59% of cases, myocardial edema. Six of the 10 patients receiving endomyocardial biopsy (EMB) were diagnosed with active lymphocytic myocarditis and two with giant cell myocarditis (GCM), while one was diagnosed with insufficient EMB. In one case, dilated cardiomyopathy (DCM) was identified histologically along with inflammatory symptoms but no myocyte necrosis. When myocarditis was first suspected in all cases, mesalamine therapy was stopped if it was already in progress. Despite receiving combined immunosuppressive therapy, two patients with GCM received heart transplants (HTx), and one patient passed away from a malignant non-cardiac cause. The median follow-up period was 24.9 months. Five patients (12%) experienced a recurrence of myocarditis. The overall results including demographic features, clinical presentations, and investigative methods used in this study are summarized in Table 5. 

**Table 5:** Clinical, imaging, and histological data of the SRL patients (N=43). *Data available data in 40 patients ~Data available data in 39 patients <Data available data in 31 patients #Data available data in 30 patients ^Data available data in 27 patients @Data available data in 22 patients >Data available data in 10 patients SRL: systematic review of literature; CD: Crohn's disease; CK-MB: creatine kinase-myocardial band; CMV: cytomegalovirus; CMR: cardiac magnetic resonance; CRP: C-reactive protein; DCM: dilated cardiomyopathy; EBV: Epstein-Barr virus; EMB: endomyocardial biopsy; ESR: erythrocyte sedimentation rate; GCM: giant cell myocarditis; IBD: inflammatory bowel disease; IQR: interquartile range; LGE: late gadolinium enhancement; LVEF: left ventricular ejection fraction; LVSD: left ventricular systolic dysfunction; NT-proBNP: N-terminal pro-brain natriuretic peptide; PBV19: parvovirus B19; UC: ulcerative colitis; WBC: white blood cell

Demographic features (N=43)	N (%)
Sex, n (%)	
Male, n (%)	33 (77%)
Female, n (%)	10 (23%)
Race, n (%)	
Caucasian, n (%)	23 (53%)
Asian, n (%)	2 (5%)
Not mentioned, n (%)	18 (42%)
Age at myocarditis diagnosis, years, median (IQR)	31 (19-44)
IBD type, n (%)	
UC, n (%)	25 (58%)
CD, n (%)	13 (30%)
Not specified, n (%)	5 (12%)
Mesalazine therapy~, n (%)	33 (85%)
Start of mesalazine <30 days from myocarditis onset, n (%)	27 (82%)
Clinical features	
Infract-like, n (%)	29 (67%)
Heart failure, n (%)	6 (14%)
Arrhythmic, n (%)	6 (14%)
No cardiac symptoms, n (%)	2 (5%)
Biochemical parameters	
Troponin I elevation*, n (%)	33 (82.5%)
WBC elevation, n (%)	10 (23%)
CRP elevation#, n (%)	25 (83%)
ESR elevation, n (%)	5 (12%)
NT-proBNP, n (%)	5 (12%)
CK-MB, n (%)	6 (14%)
Electrocardiographic features	
Sinus rhythm, n (%)	28 (65%)
Sinus tachycardia@, n (%)	10 (45%)
ST elevation@, n (%)	7 (16%)
Echocardiographic features	
LVEF, %, median (IQR)	45 (31-66)
LVSD, n (%)	15 (35%)
CMR, n (%)	33 (77%)
Edema^, n (%)	16 (59%)
LGE	24 (77%)
EMB, n (%)	10 (23%)
Lymphocytic>, n (%)	6 (60%)
GCM>, n (%)	2 (20%)
Inadequate>, n (%)	1 (10%)
DCM with sign of inflammation without myocyte necrosis>, n (%)	1 (10%)
Virology	
EBV positive, n (%)	3 (7%)
CMV, n (%)	1 (2%)
PBV19, n (%)	2 (5%)
Duration of follow-up, months, mean (IQR)	24.9 (1-71.9)
Myocarditis relapse, n (%)	5 (12%)
Death, n (%)	1 (2%)

Discussion

Pathogenesis

**Figure 2:**
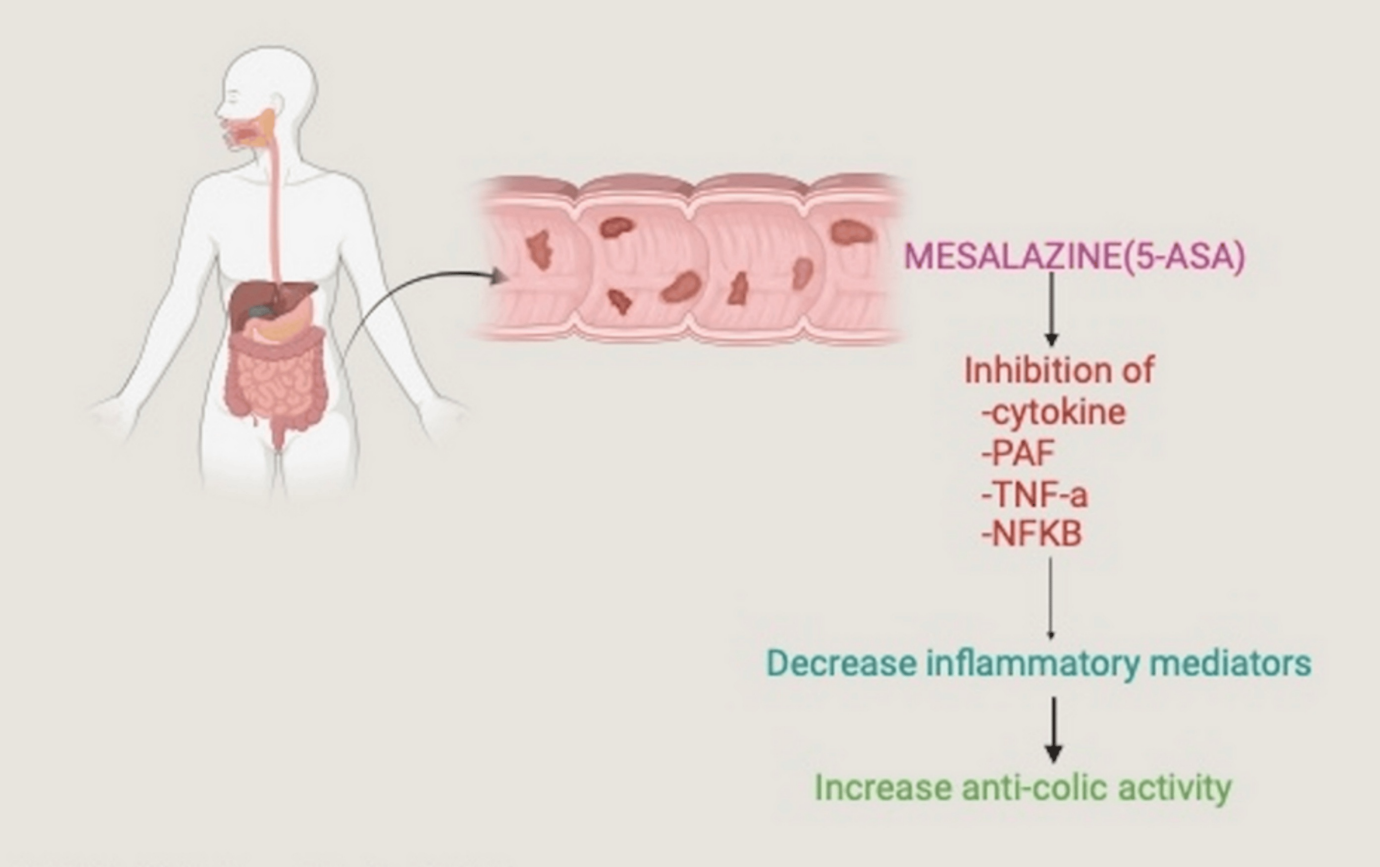
Mechanism of action of mesalazine in IBD. Image Credits: Kiran Jhakri 5-ASA: 5-aminosalicylic acid; NFKB: nuclear factor kappa light chain enhancer of activated B cells; PAF: platelet-activating factor; TNF-a: tumor necrosis factor alpha; IBD: inflammatory bowel disease

This study diagnosed 43 patients through a comprehensive evaluation of published case reports and review articles, with 22 from individual case reports and 21 sourced from a single-center cohort done by Giordani et al. in their systematic review. Mesalazine-triggered myocarditis was identified in 29 (67%) of these patients, comprising 13 from the Giordani et al. systematic review and 16 from the individual case reports. The remaining 14 (33%) patients did not exhibit myocarditis as a result of drug therapy. This finding suggests that, while myocarditis is a rare outcome, it occurs more frequently as an adverse reaction to treatment rather than as an extraintestinal manifestation of disease progression [2-4,6,8-10,13-28]. Mesalazine, known for its anti-inflammatory properties on colonic epithelial cells, is commonly used in the treatment of IBD [8]. Among the drug-induced cases, derivatives such as sulfasalazine, mesalamine, and balsalazide were identified as the primary causes of myocarditis [5]. Although the precise mechanism remains unclear, research indicates that mesalazine may exert its effects by inhibiting pro-inflammatory mediators, such as TNF-α, leukotrienes, reactive oxygen species (ROS), and interleukin-1 [8]. Figure 2 illustrates mesalazine's mechanism of action in IBD.

In a case presented by Ali et al., a patient developed heart failure and cardiogenic shock within a few days following myocarditis [8]. This case shows that although mesalazine is considered the primary treatment for IBD, it can have rare but serious side effects, including myocarditis, which can worsen over time with complications such as heart block, LV failure, dilated cardiomyopathy, cardiac arrest, and tachyarrhythmias [10]. The underlying mechanisms causing mesalazine-associated myocarditis include IgE-mediated allergic reactions, direct cardiac toxicity, cell-mediated hypersensitivity, or a humoral antibody response against 5-ASA derivatives [29]. Evidence suggests that mesalazine allergy is a type IV hypersensitivity reaction characterized by inflammation triggered by interactions between antigens and T cells, particularly T helper 1 (Th1) cells [30]. Type IV hypersensitivity develops in two phases: sensitization and elicitation. During the sensitization phase, antigen-presenting cells (APCs) absorb the antigens upon initial exposure, activating T cells in the nearby lymph nodes. This process produces both effector T cells and memory T cells, which respond rapidly upon subsequent antigen exposure. In the elicitation phase, when memory T cells re-encounter the antigen, they are activated by APCs, leading to inflammation that typically peaks at 48 hours [30].

EMBs showing infiltration of inflammatory cells consisting of lymphocytes, histiocytes, and eosinophils in a case presented by Lee and Baek support the hypothesis that mesalazine-induced cardiac inflammation is a cell-mediated hypersensitivity reaction rather than direct cardiac toxicity [15]. In the case presented by Fleming et al., the temporal association of a young patient, who was otherwise healthy and fit, developing fast progressive and severe cardiomyopathy with subsequent cardiogenic shock shortly after starting mesalazine suggests a significant correlation between the illness process and treatment, raising the possibility of drug hypersensitivity [22]. Further evidence of mesalazine hypersensitivity includes rare instances of hypersensitivity pneumonitis, angioedema, skin rashes, and hypereosinophilia [4]. Additionally, mesalazine-induced myocarditis is thought to be a humoral-mediated hypersensitivity reaction, wherein antibodies produced against mesalazine react with heart tissues to cause inflammation [31]. Mesalazine has also been shown to cause ROS to form, which harms the membrane of the mitochondria. This damage causes mitochondrial malfunction and the release of cytochrome c, which ultimately leads to cardiomyocyte death and circulatory failure [4]. The exact pathophysiology of mesalazine-induced myocarditis is yet unknown despite these hypothesized causes. The precise mechanism by which 5-ASA and its derivatives cause myocarditis in the vast majority of the patients in our study also remains unclear, indicating that this is still a difficult topic for future researchers and that further research is necessary.

Diagnosis

Diagnosing myocarditis can be challenging due to the variety of clinical manifestations. In most patients reviewed, the diagnosis of mesalazine-induced myocarditis has been established based on laboratory results, clinical characteristics, and the duration of symptoms since the start of therapy [2-4,6,8-10,13-28]. Typically, patients with mesalazine-induced myocarditis experience symptoms within two weeks of treatment initiation, whereas myocarditis as an extraintestinal manifestation occurs during the acute exacerbation of IBD [19,21,23,24,26,27,29]. About 82% of patients in our study experienced symptoms of myocarditis within 30 days after the initiation of mesalazine therapy. The instance provided by Fleming et al. in our study had the earliest symptom onset, occurring three days after therapy began [22]. However, the patient in the case described by Okoro et al. exhibited symptoms six months later. This delayed onset of myocarditis is thought to be related to the coadministration of steroids and mesalamine [16].

Myocarditis can manifest with various symptoms, such as arrhythmias, cardiogenic shock, acute coronary syndrome, decompensated or new-onset heart failure, or sudden death [29]. This study found that 67% of participants experienced infarct-like symptoms, 14% had heart failure symptoms, and 14% presented with arrhythmia symptoms [2-4,6,8-10,13-28]. These findings align with the higher prevalence of infarct-like myocarditis reported in the general population [29]. Of note, two patients had no cardiac symptoms; diagnosis was made based on changes in biochemical and other investigations [6,24].

Determining the true prevalence of myocarditis is difficult because EMB, which is considered the gold standard for diagnosis, is rarely performed [7]. Only 23% of the patients did EMB in our study [6,15]. It is still the most reliable method for identifying myocarditis, although it is rarely carried out. Based on clinical presentations and diagnostic techniques such as CMR, cardiac biomarkers, transthoracic echocardiography (TTE), and electrocardiogram (ECG), noninvasive diagnostic criteria have been developed for instances clinically suspected of myocarditis [27]. Nowadays, CMR is considered the first-line noninvasive technique for evaluating patients suspected of having myocarditis [7]. CMR was done in the majority of our patients (77%). It can show localized fibrosis or necrosis without a coronary arterial distribution, as well as myocardial edema and hyperemia, whether regional or global, in patients diagnosed with myocarditis [29]. CMR in our study showed LGE in 77% and edema in 59% of the patients. CMR has proven effective for assessing myocardial inflammation and damage in myocarditis and plays a vital role in diagnosing and monitoring the disease's progression. The most recent position paper on myocarditis from the European Society of Cardiology highlights its critical importance [13]. 

An ECG may show abnormalities or changes in the ST segment and T wave. In our study, 65% had sinus rhythm, 45% had sinus tachycardia, and 16% had ST elevation [2-4,6,8-10,13-28]. Laboratory tests may indicate leucocytosis, increased erythrocyte sedimentation rate (ESR), elevated CRP, and increased levels of cardiac biomarkers such as troponin I, creatine kinase-myocardial band (CK-MB), brain natriuretic peptide (BNP), and N-terminal pro-brain natriuretic peptide (NT-proBNP) [29]. Most of the patients in our review had elevated troponin I (82.5%) and CRP (83%), whereas only a few had elevated white blood cell (WBC) (23%), ESR (12%), NT-proBNP (12%), and CK-MB (14%) [2-4,6,8-10,13-28]. TTE also played a significant role in diagnosing myocarditis. Myocarditis may present on TTE as pericardial effusion, reduced ejection fraction, abnormalities in regional wall motion, or LV failure [29]. In our study, the median LVEF was 45% (IQR 31-66), and left ventricular systolic dysfunction (LVSD) was found in 35% of the patients, whereas normal reports were found in 9% of the patients [4,17,20,27,28]. Computed tomography angiography (CTA) was done in the majority of cases to rule out pulmonary embolism. Song and Seo presented a case where the patient had pulmonary edema and pleural effusion found on the chest CT along with cardiac symptoms [3]. This shows that while suspecting diagnosis, it is very important to rule out ongoing pulmonary cause. 

Management

Discontinuing the causative medication has been the cornerstone of treatment in most cases analyzed in our study. Around 87% of the patients had complete resolution of symptoms after withdrawal of drugs, whereas relapse occurred in 12% of the patients. In most patients, symptoms resolved within two weeks of stopping the medication, indicating a relatively swift recovery period. The symptoms in the one reported by Ibrahim et al. and Baker et al. resolved fastest within 48 hours and four days, respectively, as opposed to the typical 1-2 weeks. This quicker recuperation could be because the patient received corticosteroid therapy simultaneously, which probably helped control inflammation [18,20]. However, in a case reported by Caio et al., myocarditis symptoms recurred even after mesalazine had been discontinued for a year. This case underscores the need to remain vigilant about the possibility of myocarditis as an extraintestinal manifestation related to disease flare-ups and increased intestinal permeability [14]. It draws attention to how difficult it can be to differentiate between adverse drug reactions and symptoms of the underlying IBD [14]. 

After the withdrawal of mesalazine, immunosuppressive therapies such as corticosteroids, azathioprine (AZA), cyclosporine, and 6-mercaptopurine were started in most of the patients and became an essential part of the therapeutic process. Gruenhagen et al. described a case where mesalamine and corticosteroids were used to treat both the intestinal symptoms of IBD and myocarditis [19]. This dual-purpose approach illustrates the overlap between treatments for IBD and myocarditis, given that immunosuppressive therapies or immunoglobulins are commonly employed for inflammatory myocarditis. Since these therapies are also standard treatments for IBD, in many cases, no specific modifications to the therapeutic regimen are necessary [19]. As stated in publications by Baker et al. and Okoro et al., immunosuppressants not only reduced the amount of time needed for symptoms to go away, but they might have even prevented some patients' symptoms from starting in the first place [16,18]. However, because myocarditis might reoccur, close monitoring of the patient's condition (if there are any withdrawal symptoms such as fever, nausea, vomiting, and low blood pressure) is required throughout the period of steroid reduction. Increasing the steroid dosage is usually advised if recurrence is noticed. It is also important to rule out any viral causes or other immunosuppressive therapeutic contraindications before starting steroid treatment [16,18].

Other treatment options for myocarditis in IBD patients include nonsteroidal anti-inflammatory medications (NSAIDs) like aspirin or indomethacin [5]. Additionally, when mesalazine is contraindicated due to myocarditis, alternative long-term therapies for IBD often include biological drugs. These biologics, such as infliximab, adalimumab, vedolizumab, and filgotinib, have effectively managed inflammation and maintained disease remission in many cases [8-10,14,16,18,25,27]. 

Kim et al. reported a complex case in which extracorporeal membrane oxygenation (ECMO) was successfully employed to provide cardiac support for suspected acute myocarditis associated with UC. In that instance, the addition of infliximab was necessary to control inflammation that was unresponsive to corticosteroids [23]. This case shows the need to take a comprehensive approach to treatment when standard drugs are insufficient [23]. Current guidelines strongly advise that patients diagnosed with myocarditis refrain from participating in both competitive and recreational sports, as physical exertion can exacerbate cardiac complications [29]. To prevent potentially fatal myocarditis-related outcomes, careful monitoring of the patient's condition (vital signs like heart rate, blood pressure, respiratory rate, symptoms like chest pain, dyspnea, and any changes in cardiac biomarkers and other investigations) and a study of the patient's medical history are required. An early and accurate diagnosis can ensure the best patient outcomes and efficient management strategies.

Limitations

Several factors limit the interpretation of our findings. First, most of the included publications were case reports, which lack control groups and can introduce publication bias by preferentially highlighting severe or unusual presentations. As a result, the true incidence of mesalazine-induced myocarditis cannot be assessed. Second, our study focuses on patients aged 19-44 years at the time of diagnosis of mesalazine-induced myocarditis and excludes pediatric and older adult populations, who may have different risk profiles and treatment responses. Third, restricting our search to English-language articles and excluding unavailable full-text papers could create a language and access bias, potentially omitting relevant data from non-English-speaking regions. Fourth, potential confounders such as concurrent steroid use, the severity of IBD, and existing cardiac risk factors were not uniformly reported, limiting the ability to generalize these findings. Lastly, because EMBs are rarely conducted, milder or subclinical myocarditis cases may be missed, underrepresenting the full clinical spectrum of mesalazine-induced cardiac complications. 

## Conclusions

Myocarditis induced by mesalazine is a rare event in IBD, and its etiology and management have yet to be adequately researched, which is a challenge for clinicians. Myocarditis, while an uncommon complication in IBD, is more commonly an adverse side effect of medical therapy rather than an extraintestinal manifestation. Therefore, thorough investigation and regular monitoring of the patient's medical history are important to prevent potentially fatal consequences associated with myocarditis. The most common cause of drug-induced myocarditis is the use of 5-ASA derivatives, such as sulfasalazine, mesalamine, and balsalazide. IgE-mediated allergy reactions, direct cardiac toxicity, cell-mediated hypersensitivity, and humoral antibody response to 5-ASA derivatives are the underlying mechanisms that cause mesalamine-associated myocarditis. A diagnosis can be established based on laboratory results, clinical features, and the duration of symptoms after treatment. Most people react well to stopping their medications and receiving immunosuppressive therapies. Early diagnosis enables the delivery of the best possible patient care. More research is needed to establish how to differentiate extraintestinal symptoms of IBD from drug-induced myocarditis. To prevent and treat IBD, it is also important to research the risk factors for myocarditis development in patients with the disease.
